# A Rapid and Highly Efficient Method for Transient Gene Expression in Rice Plants

**DOI:** 10.3389/fpls.2020.584011

**Published:** 2020-10-15

**Authors:** Naini Burman, Divya Chandran, Jitendra P. Khurana

**Affiliations:** ^1^Regional Centre for Biotechnology, Faridabad, India; ^2^Department of Plant Molecular Biology, University of Delhi, New Delhi, India

**Keywords:** transient expression assay, rice, BiFC analysis, localization, confocal imaging, *Agrobacterium* – mediated transformation

## Abstract

Rice is the model plant system for monocots and the sequencing of its genome has led to the identification of a vast array of genes for characterization. The tedious and time-consuming effort of raising rice transgenics has significantly delayed the pace of rice research. The lack of highly efficient transient assay protocol for rice has only added to the woes which could have otherwise helped in rapid deciphering of the functions of genes. Here, we describe a technique for efficient transient gene expression in rice seedlings. It makes use of co-cultivation of 6-day-old rice seedlings with *Agrobacterium* in the presence of a medium containing Silwet^®^ L-77, acetosyringone and glucose. Seedlings can be visualized 9 days after co-cultivation for transient expression. The use of young seedlings helps in significantly reducing the duration of the experiment and facilitates the visualization of rice cells under the microscope as young leaves are thinner than mature rice leaves. Further, growth of seedlings at low temperature, and the use of surfactant along with wounding and vacuum infiltration steps significantly increases the efficiency of this protocol and helps in bypassing the natural barriers in rice leaves, which hinders *Agrobacterium-*based transformation in this plant. This technique, therefore, provides a shorter, efficient and cost-effective way to study transient gene function in intact rice seedling without the need for a specialized device like particle gun.

## Introduction

Rice is an important cereal crop plant, which is the staple food of Asia and part of the pacific. According to FAO, there is a thin line of rice self-sufficiency in many countries and the increasing population pressure is making this line disappear fast (Sainsbury and Lomonossoff, 2014). The ultimate aim of rice research is to increase production using less land, less manpower, less water and fewer pesticides. To achieve this aim, a basic understanding of this plant is essential, which requires in depth knowledge of the functions of different genes and their interactions. Availability of high quality sequence of the rice genome and genes represented on its landscape (International Rice Genome Sequencing Program, 2005; Vij et al., 2006) opened up avenues to achieve these goals. One way to study the function of rice genes is to express them in plants through stable or transient expression (Sainsbury and Lomonossoff, 2014). Transient expression systems yield faster results as compared to stable transgenics. A transient expression system is considered ideal if it is technologically simple, cost-effective, robust, high throughput and confers high transformation efficiencies (Page et al., 2019). Transient expression is very useful for checking the intracellular localization of proteins, to study *in-vivo* protein-protein, protein-DNA and protein-RNA interactions and to perform Co-IP, ChIP and protein activity experiments. Different plant species and tissues show a lot of variability in susceptibility to *Agrobacterium*-mediated transient expression of genes (Krenek et al., 2015). While transient expression of genes in *Nicotiana benthamiana* and *Arabidopsis* is widely used to achieve the above objectives, most of the transient expression analyzes of rice genes is done either in rice protoplasts or onion epidermal cells or in *Arabidopsis* or tobacco plants. However, these heterologous systems are prone to exhibit aberrant traits (Zhang et al., 2011). Transient expression systems in rice can be used for functional characterization of single genes as well as characterization of signaling pathways and gene networks (Kim et al., 2015). Currently, majority of rice transient assays are done in rice protoplasts that do not replicate the conditions of intact rice tissue (Marx, 2016). In addition, the short life span of protoplasts and the expensive enzymes utilized for its production makes the utility of protoplasts for the above experiments less attractive. Therefore, the need of the hour for rice research is the development of an efficient, rapid and cost-effective transient gene expression protocol but the structure of rice leaves with high silica content and epicuticular wax has been a major obstacle in achieving this goal. Even after more than 15 years of establishment of rice transgenics protocols (Toki et al., 2006), very few in-planta rice protein localization protocols are available and that too with very low transient transformation efficiencies (Luginbuehl et al., 2020). While in many plant species, fluorescent reporters are widely used for localization in all major organs, in case of rice, fluorescent reporters have been used to study only roots and non-green tissues of the plant. Most of the studies involving green tissues and fluorescent reporters have been done in leaf sheath and only few reports exist for rice leaves. The thick waxy cuticle of rice leaves is known to scatter light, thereby, impeding high resolution fluorescent protein visualization (Luginbuehl et al., 2020). The presence of epicuticular wax on rice leaves also makes transient expression of genes using *Agrobacterium* difficult (Sharma et al., 2020). Here, we present a highly efficient and rapid protocol for transient expression of genes in rice seedlings without the requirement of high-end equipment. We were able to overcome the structural barriers (high epicuticular wax content) for transformation of rice leaves by *Agrobacterium* by growing the seedlings at low temperature.

## Materials and Equipment

1. *Agrobacterium* EHA105 strain
2. Freshly harvested Kitaake rice seeds
3. In-house designed plant growth chamber (Yatherm)
4. 0.1% HgCl_2_ (w/v)
5. Milli-Q water
6. Translucent phyta-jar (HiMedia # PW1138)
7. Whatman^®^ No. 1 filter paper
8. ½ inch 3M Micropore tape
9. pSITE-2CA vector (1 μg)
10. pCAMBIA1304 (1 μg)
11. pCAMBIA1302 (1 μg)
12. pSITE-nEYFP-C1 with *OsbZIP48* cloned (1 μg)
13. pSITE-nEYFP-N1 with *OsbZIP48* cloned (1 μg)
14. Sorvall SS-34 tubes
15. 50 ml Luria Broth (LB) medium
16. 250 ml Vacuum flask with cork -2
17. Horizontal laminar flow (Esco Airstream^®^)
18. 60 ml co-cultivation medium
19. 18 Ga needle
20. Vacuum pump
21. 4 μg/ml DAPI (Sigma)
22. Leica True Confocal Scanning-Spectral Photometric 5 II microscope
23. Leica Stereo microscope (M205C)
24. Coverslips
25. Glass slides
26. 2 ml Micro centrifuge tubes (MCTs) (Tarsons)
27. 100% glycerol (Sigma)
28. Corning 96-well plate, flat bottom, black polystyrene (#CLS3925)
29. Spectramax^®^ i3x Minimax^TM^ 300 imaging cytometer (Molecular devices)
30. DAPI staining medium- 1xPBS, 0.02% Silwet^®^ L-77 (v/v), 0.02% DMSO (v/v), 4 μg/ml DAPI
31. Bradford solution (Himedia)
32. Anti-GFP antibody (# G1544, Sigma)
33. Anti-Actin antibody (#SAB4301137, Sigma)
34. **Composition of Co-cultivation medium**

**Table:** 

Chemicals	Amount (for 500 ml)
NB medium (Himedia, # PT107)	12.07 g
L-Proline (Duchefa)	1.435 g
L-Glutamine (Duchefa)	0.25 g
Caesin enzyme hydrosylate Type-1 (Himedia)	0.15 g
Sucrose (Qualigens)	5 g (in addition to 20 g/L already present in commercially available NB medium)
D-(+)-Glucose (Sigma)	10 g
Acetosyringone (Himedia)	30 mg/L (Dissolved in methanol; freshly prepared)
Silwet^®^ L-77 (Lehle seeds)	0.02% v/v
Add water to make up the volume and set pH to 5.2 by 1 M KOH
35. **Buffers required for GUS assay**

**Table:** 

Chemicals	Stock solution	Working concentration	Amount
**Protein extraction buffer**			**For 10 ml**
Tris-Cl buffer (pH 8) (Bio-rad)	1 M	200 mM	2 ml
NaCl (Sigma)	1 M	100 mM	1 ml
Sucrose (Qualigens)	-	400 mM	1.369 g
EDTA, pH 8 (SRL Diagnostics)	1 M	10 mM	100 μl

**Table:** 

Chemicals	Stock solution	Working concentration	Amount
DTT (Himedia)	-	10 mM	15.42 mg
Plant protease inhibitor cocktail (Sigma)	-	1%	100 μl (to be freshly added)
Tween 20 (Bio-rad)	-	0.05%	5 μl
PMSF (Sigma)	100 mM	1 mM	100 μl (to be freshly added)
Glycerol (Sigma)	-	5%	500 μl
Milli-Q water			To make up the volume
**GUS assay reaction buffer**			**For 10 ml**
EDTA, pH 8 (SRL diagnostics)	1 M	10 mM	100 μl
Sodium dodecyl sulfate (SDS) (Sigma)	10%	0.1%	100 μl
Sodium phosphate buffer, pH 7 (Sigma)	1 M	50 mM	500 μl
Triton X-100 (Sigma)	-	0.1%	10 μl
4-Methylumbelliferyl-β-D-glucuronide hydrate or MUG (Sigma)	-	0.6 mM	2.1 mg
β-Mercaptoethanol (Gibco)	-	12 mM	8.3 μl
Water			To make up the volume
**Stop buffer**			**For 10 ml**
Sodium carbonate (Sigma)	-	0.2 M	212 mg in Milli-Q
36. **GUS Histochemical staining buffer**

**Table:** 

Chemicals	Stock solution	Working concentration	Amount (for 10 ml)
Sodium Phosphate buffer	1 M	50 mM	500 μl
Potassium ferrocyanide	1 M	0.2 mM	2 μl
Potassium ferricyanide	1 M	0.2 mM	2 μl
Triton X-100		0.2%	20 μl
X-Gluc (Himedia) (Dissolved in DMSO)		1 mM	4.4 mg
Milli-Q water			To make up the volume
37. **1X Phosphate saline solution (PBS) (pH 7.4)**

**Table:** 

Chemical	Final concentration	Amount to be added
NaCL (Sigma)	137 mM	8.0 g
KCl (Sigma)	2.7 mM	0.2 g
Na_2_HPO_4_ (Sigma)	10 mM	1.44 g
KH_2_PO_4_ (Sigma)	1.8 mM	0.24 g

## Materials and Methods

### Plant Growth Conditions

1. Freshly harvested seeds of rice japonica variety, Kitaake, were surface sterilized inside the laminar flow with 0.1% HgCl_2_ (with 0.002% Teepol) for 10 min, washed repeatedly with autoclaved Milli-Q water and then kept at 28 ± 1°C for 48 h for imbibition in dark.
2. The seeds were then placed in translucent phyta-jar (HiMedia # PW1138) layered with a sheet of autoclaved Whatman^®^ No. 1 paper at the base moistened with 4 ml of autoclaved Milli-Q water.
3. The phyta-jars were sealed with micropore tape and kept in a growth chamber at 20 ± 1°C under 12 h light/12 h dark conditions.
4. For study of developmental pattern under different conditions, the length of different tissues like coleoptile and first leaf were measured from day 4 to day 11 for seedlings grown at 20°C and from day 3 to day 10 for those grown at 22°C. Day 1 is considered from 24.01 to 48 h after keeping the seeds at 20°C and 22°C. 6-day-old seedlings were photographed using a Leica stereo microscope.

### *Agrobacterium*-Based Seedling Transformation

1. The pSITE-2CA vector (for GFP) (Chakrabarty et al., 2007) and pCAMBIA1304 (for GUS) vectors were transformed in *Agrobacterium* cells (strain EHA105) by chemical transformation method.
2. The resulting colonies were screened for the presence of the plasmid and then used for transformation.
3. A day before co-cultivation, the *Agrobacterium* colony was inoculated in 50 ml of liquid LB (Luria broth) medium with desired antibiotics (containing 30 μg/ml rifampicin and 100 μg/ml spectinomycin for pSITE-2CA; 30 μg/ml rifampicin and 50 μg/ml kanamycin for pCAMBIA1304) and allowed to grow at 28°C, 200 rpm.
4. After 12–18 h, the culture was pelleted down in a high-speed centrifuge (Sorvall; SS 34 rotor) by centrifuging at 4000 rpm, at 25°C for 30 min.
5. The *Agrobacterium* pellet was resuspended in 10 ml of co-cultivation medium depending on the pellet size.
6. The transformation process was carried out in the Laminar air flow cabinet (Esco) using sterile glassware and media. In an autoclaved vacuum flask, 50 ml of co-cultivation medium was poured and *Agrobacterium* cells solution was added so that the final A_600_ of *Agrobacterium* in the co-cultivation medium was 0.6.
7. Six-day-old rice seedlings were taken out from the phyta-jar and the first leaf of the seedling was wounded by pricking with a needle (18 Ga needle used in 10 ml syringe) three times along the length of the first leaf.
8. The seedlings (about 50 to 60) were then transferred in the vacuum flask containing 50 ml of the co-cultivation medium. The mouth and sprout of the vacuum flask was covered with an aluminum foil and kept in an incubator shaker at 28°C, 60–90 rpm for 1 h so that the seedlings swirl gently.
9. After 1 h, the flask was attached to a vacuum pump for 15 min at 500 mm Hg. The solution was decanted and the seedlings were put on autoclaved tissue paper and then put back in the phyta-jars with autoclaved Whatman^®^ No.1 filter paper sheets as the base.
10. Prior to placing the seedlings, the Whatman^®^ No.1 filter paper sheets placed in the phyta-jar were soaked with 2–3 ml of autoclaved Milli-Q water.
11. The phyta-jars were sealed with micropore tape and then placed in a growth chamber at 20 ± 1°C under 12 h light/12 h dark conditions for 9 days.

### BiFc Analysis

1. For BiFc assay, full length CDS of *OsbZIP48* was cloned into pSITE-nEYFP-C1 and pSITE-nEYFP-N1 vectors. The vector map of these vectors has been provided in Supplementary Figure 1. The N-terminal half of YFP is present in pSITE-nEYFP-N1 vector and the C-terminal half of YFP is present in pSITE-nEYFP-C1. If OsbZIP48 forms a homodimer, then both halves of YFP will come together to form a full YFP protein and emit fluorescence (Martin et al., 2009).
2. The constructs were transformed into *Agrobacterium* EHA105 strain separately. Each *Agrobacterium* strain transformed with the above mentioned constructs was then inoculated in 50 ml LB medium (containing 30 μg/ml rifampicin and 100 μg/ml spectinomycin) each. Each culture was pelleted separately at 4500 rpm for 30 min at 25°C and the pellet was re-suspended in 10 ml of co-cultivation medium. The co-cultivation was carried out in the same way as described above except A_600_ of each construct containing *Agrobacterium* was 0.3.

### Live Cell Imaging

1. After 9 days, the putatively transformed seedlings were taken out of the phyta-jar and washed with RO water 3–5 times.
2. The first leaf of the seedlings was picked up using forceps.
3. The first leaf from seedlings were placed in 2 ml MCT containing DAPI staining medium and kept in a desiccator attached to vacuum pump for 10 min and then washed with RO water for 5 min, with mild shaking.
4. The first leaf was mounted on glass slides using mounting medium (1 ml RO water + 3–4 drops glycerol).
5. The coverslip was sealed on all sides using tape. The slide was then visualized under Leica True Confocal Scanning-Spectral Photometric 5 II microscope. For GFP, excitation at 488 nm and emission range of 498 nm to 530 nm was used and for YFP, excitation at 514 nm and emission range of 524 to 546 nm was used.

### Total Protein Extraction and Fluorescent β-Galactosidase Assay

1. The washed first leaves were ground in liquid nitrogen and re-suspended in 750 μl of protein extraction buffer on ice.
2. The tissue sample was centrifuged at 13,000 rpm, 4°C for 45 min. The supernatant was again centrifuged at 13,000 rpm, 4°C for 45 min.
3. This protein extract was then quantified by Bradford assay (Bradford, 1976). The assay was performed directly in 96-well microtiter plates.
4. GUS assay reaction buffer was prepared and 50 μl was added to each well having the appropriate volume of plant extracts containing 2 μg total protein.
5. The plates were incubated for 4 h at 37°C in an incubator, in dark. The reactions were stopped by adding 150 μl stop buffer (0.2 M Na_2_CO_3_) to each well and mixed well.
6. The samples were measured in a microplate fluorimeter (Spectramax i3x) with the following settings: excitation filter at 365 nm, emission filter at 460 nm, gain = low and number of flashes set at 25.
7. The data was plotted using GraphPad and statistical analyses were performed using unpaired *t*-test and one-way anova using GraphPad.

### GUS Histochemical Assay

9-day-old rice seedlings were immersed in GUS histochemical buffer and vacuum treatment was applied to facilitate the penetration of the staining solution. The staining was carried out at 37°C in the dark for 48 h. The chlorophyll was removed by extensive destaining with 100% ethanol.

### Western Blotting

Frozen first leaves of seedlings transformed with pCAMBIA1302 vector were ground in a buffer (200 mM Tris, pH 8, 100 mM NaCl, 10 mM EDTA, 10 mM DTT, 5% glycerol, 0.05% Tween 20, and protease inhibitor cocktail [Sigma]), and the extracts were centrifuged at 13,000 rpm and 4°C for 30 min. The supernatant was centrifuged again at 13,000 rpm and 4°C for 15 min and the concentration of proteins was estimated by the Bradford assay (Bradford, 1976). Equal concentrations of proteins were loaded on SDS-PAGE gel, and the protein extracts were subjected to western blotting (see Burman et al., 2018). For detection of GFP protein levels, anti-GFP antibody (# G1544, Sigma) was used while for detection of actin levels (used as loading control), anti-actin antibody (#SAB4301137, Sigma) was used.

## Results

### Optimization of Growth Conditions and Identification of Suitable Developmental Stage for Transient Gene Expression

**FIGURE 1:**
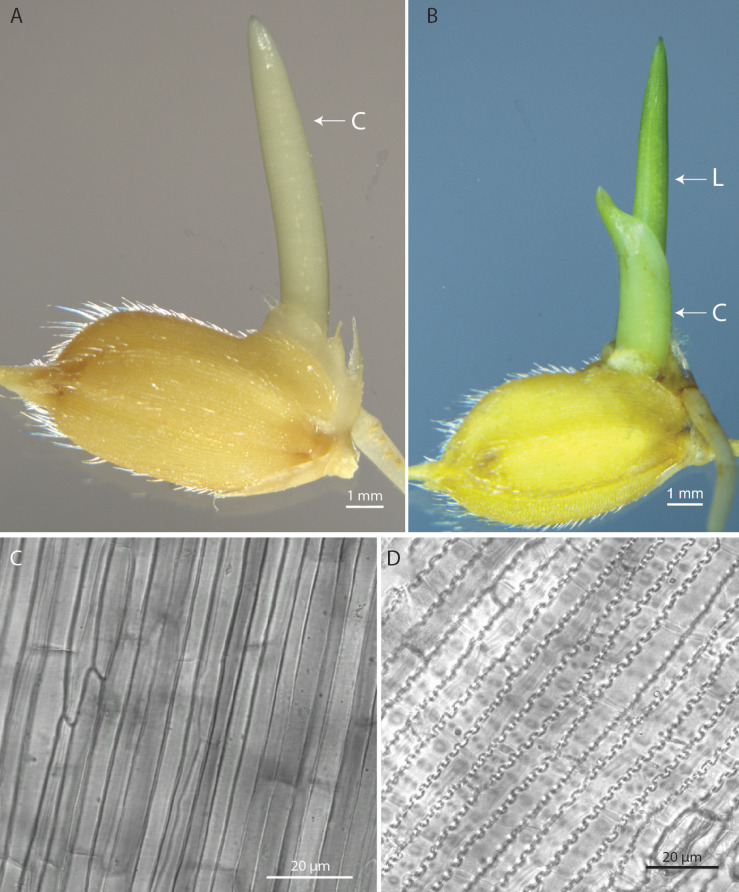
Morphology of rice seedlings and cells. **(A)** 4-d-old dark-grown seedling. **(B)** 6-d-old light-grown seedling. **(C)** Morphology of coleoptile epidermal cells. **(D)** Morphology of first leaf epidermal cells. Scale bar = 1 mm (For **A** and **B**), Scale bar = 20 μm (for **C** and **D**). C, Coleoptile; L, First leaf.

To identify the optimal growth temperature, rice seeds were grown at two temperatures- 20°C and 22°C. The main reason for using low temperature was to reduce the formation of epicuticular wax as it is controlled by temperature (Go et al., 2014). The initial stages of rice seedling growth are marked with the emergence of coleoptile and root followed by the emergence of first leaf and second leaf. The emergence and survival time for these different tissues is light- and temperature-dependent. To have a time-efficient protocol, the rice seedling part to be used for transient experiments should emerge within 1 week and should stay alive for a reasonable number of days for the transformed gene to express in sufficient quantity. The two shoot tissues which showed early emergence included coleoptile and the first leaf (Figures 1A,B). For this reason, coleoptile and first leaf were selected for transformation in dark-grown and light-grown seedlings, respectively (Figure 1). As shown in Figures 1C,D, the cells of coleoptile of 4-d-old dark-grown seedling exhibit greater similarity to onion epidermal cells than the 6-d-old light-grown first leaf cells, and are devoid of chlorophyll indicating that they are a better candidate for protein localization studies. A time-course experiment to study the development pattern of these two tissues was done at 20°C and 22°C (Supplementary Table 1). The coleoptile and first leaf length and the emergence percentage of the first leaf were measured in 3-d- to 10-d-old seedlings grown in dark and light at 22°C and in 4-d- to 11-d-old seedlings grown in dark and light at 20°C (Supplementary Table 1). In dark-grown seedlings grown at 20°C, the coleoptile started to emerge from 4-d onwards and reached its maximum length at 9-d while the first leaf started to emerge on 11-d after germination. In light-grown seedlings grown at 20°C, the coleoptile length reached its maximum on 7-d after germination while the first leaf began to emerge on day 5 and continued to grow till day 11. At day 6, approximately, 60% of the rice seedlings had their first leaf emerged. In dark-grown seedlings grown at 22°C, the coleoptile reached its full growth at 9-d after germination. The first leaf emerged at 7-d after germination. In light-grown seedlings raised at 22°C, coleoptile reached its full growth at 5-d after germination while the first leaf emerged at 4-d after germination. At 5-d after germination, around 80% of total seedlings showed first leaf emergence. Based on these observations, 4-d-old seedlings grown in dark at 20°C and 22°C and 6-d-old and 5-d-old seedlings grown in light at 20°C and 22°C, respectively, were chosen for *Agrobacterium*-mediated transformation.

### Standardization of the Transient Assay

**FIGURE 2:**
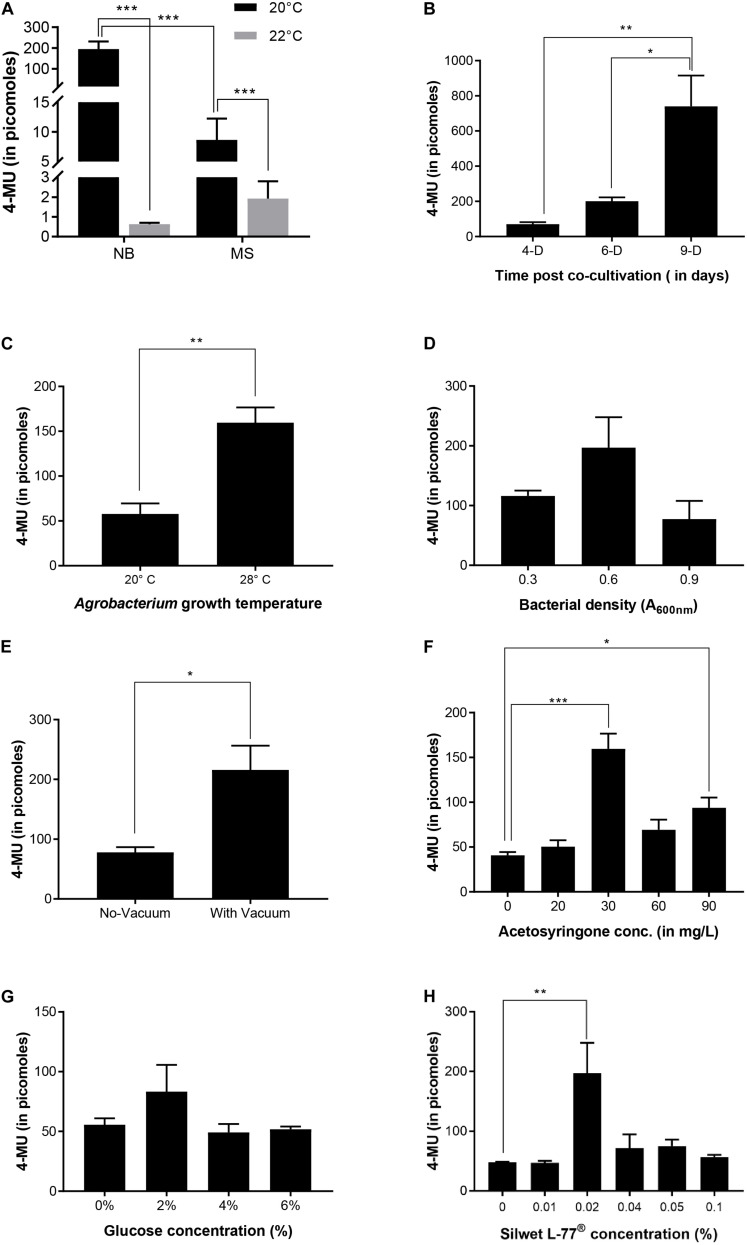
Optimization of transient expression in rice. **(A)** Effect of co-cultivation medium and seedling growth temperature on transient expression. **(B)** Time-course for transient expression. Transient expression efficiency was quantified by the measuring 4-MU fluorescence of total protein extract isolated from the first leaf of rice seedlings at different time points during the co-cultivation when 0.02% Silwet^®^ L-77 and bacteria of A_600__nm_ = 0.6 were used in the co-cultivation medium. **(C)** Effect of *Agrobacterium* growth temperature on transient expression. *Agrobacterium* strain transformed by pCAMBIA1304 was grown in LB medium with antibiotics at temperatures mentioned in the graph and 1 h co-cultivation in the medium with shaking was also carried out at the same temperature. **(D)** Effect of bacterial density on transient expression efficiency. **(E)** Effect of vacuum pressure on transient expression efficiency. **(F)** Effect of acetosyringone concentration on transient expression efficiency. **(G)** Effect of glucose concentration on transient expression efficiency. **(H)** Effect of Silwet^®^ L-77 concentration on transient expression. *n* = 3 biological replicates (mean ± standard error for **A–H**). ***p-value ≤ 0.001, **p-value ≤ 0.01, *p-value ≤ 0.05.

To select the appropriate medium, temperature, light condition and post-co-cultivation visualization day, two commonly used rice co-cultivation media were used for the transient assay. These were MS (Murashige and Skoog, 1962) medium and NB medium (Chu et al., 1975). The rice seedlings were grown at 20°C and 22°C in presence of light and the MUG fluorescence was measured at 9-d after co-cultivation for light-grown seedlings (Figure 2A). We found that NB medium was better than MS medium as co-cultivation medium for the seedlings grown at 20°C in light. Transformation of dark-grown coleoptile was also performed and the cells visualized at 7-d after co-cultivation but the transformation efficiency was very low (data not shown). Time post-co-cultivation was standardized with MUG fluorescence reading taken at 4-d, 6-d and 9-d after co-cultivation (Figure 2B). It was found that fluorescence started increasing from 4-d after co-cultivation and was maximum at 9-d after co-cultivation indicating that the best time for GFP visualization in rice cells is 9 days after co-cultivation. Tissues after 9-d post-co-cultivation were not used for fluorescence quantification as the first leaf showed signs of senescence after this period.

**FIGURE 3:**
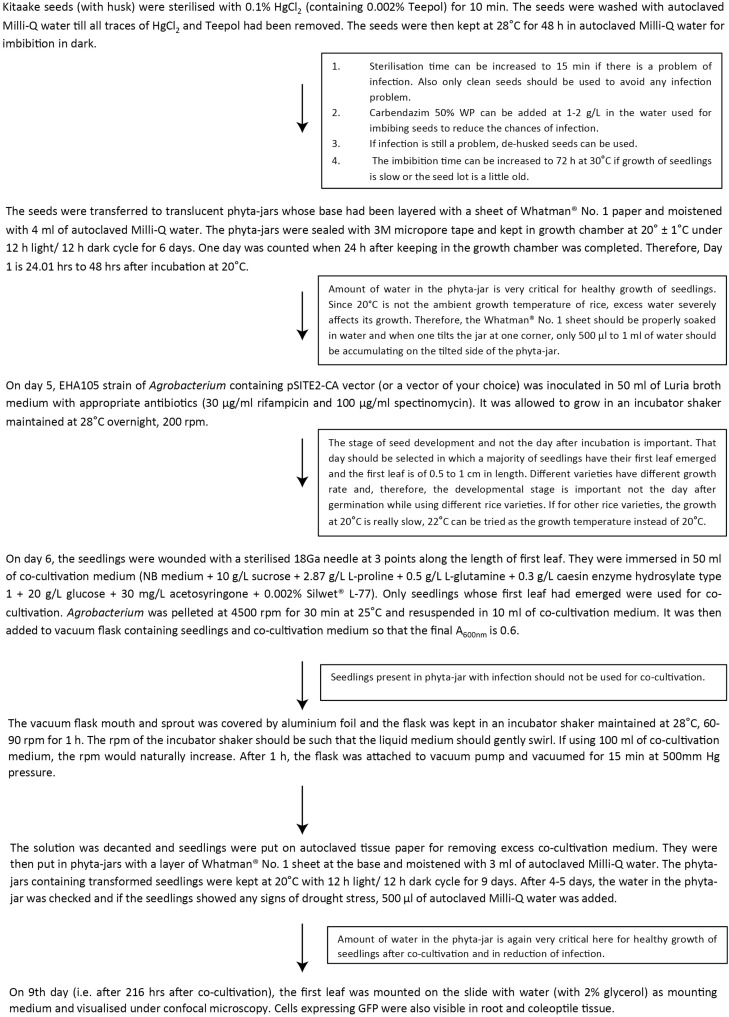
Step by step protocol used for transient expression in rice seedlings.

**FIGURE 4:**
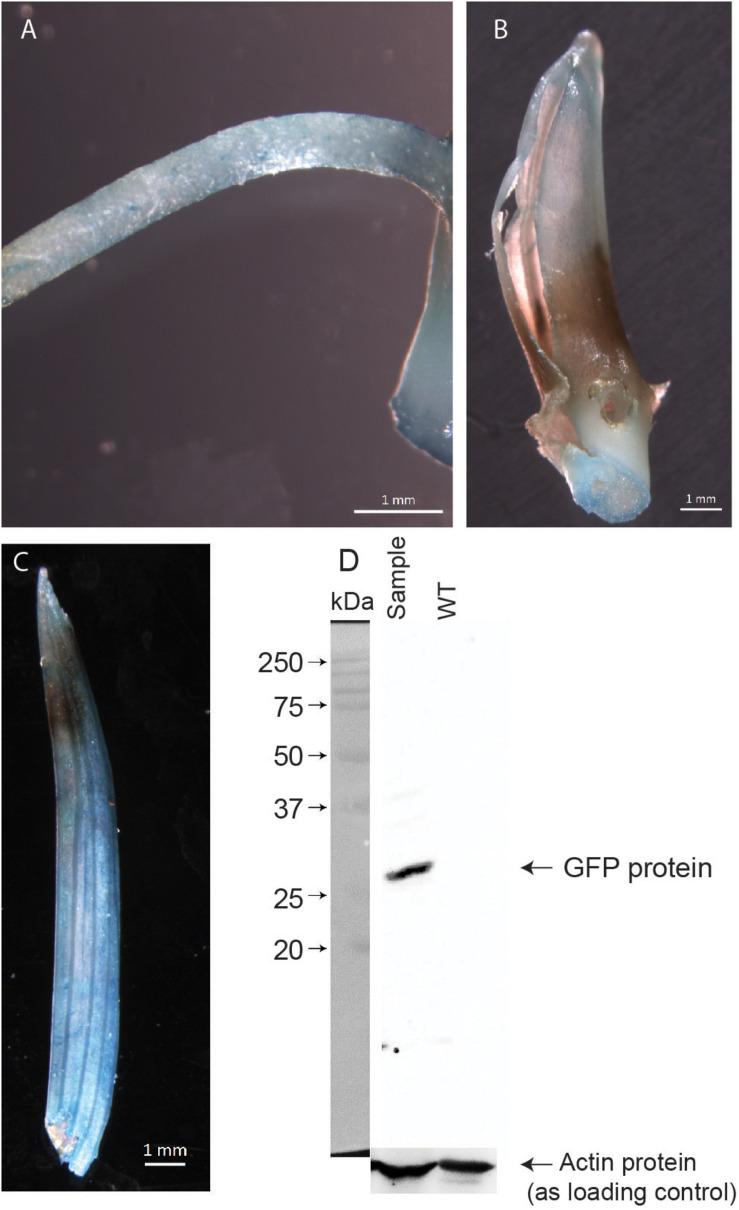
GUS histochemical assay of transformed rice seedlings. **(A)** Root of the transformed rice seedling. **(B)** Coleoptile of the transformed rice seedling. **(C)** First leaf of the transformed rice seedling. **(D)** Western blot showing GFP protein level in the protein extracts of first leaf of rice. Actin protein levels used as loading control.

**FIGURE 5:**
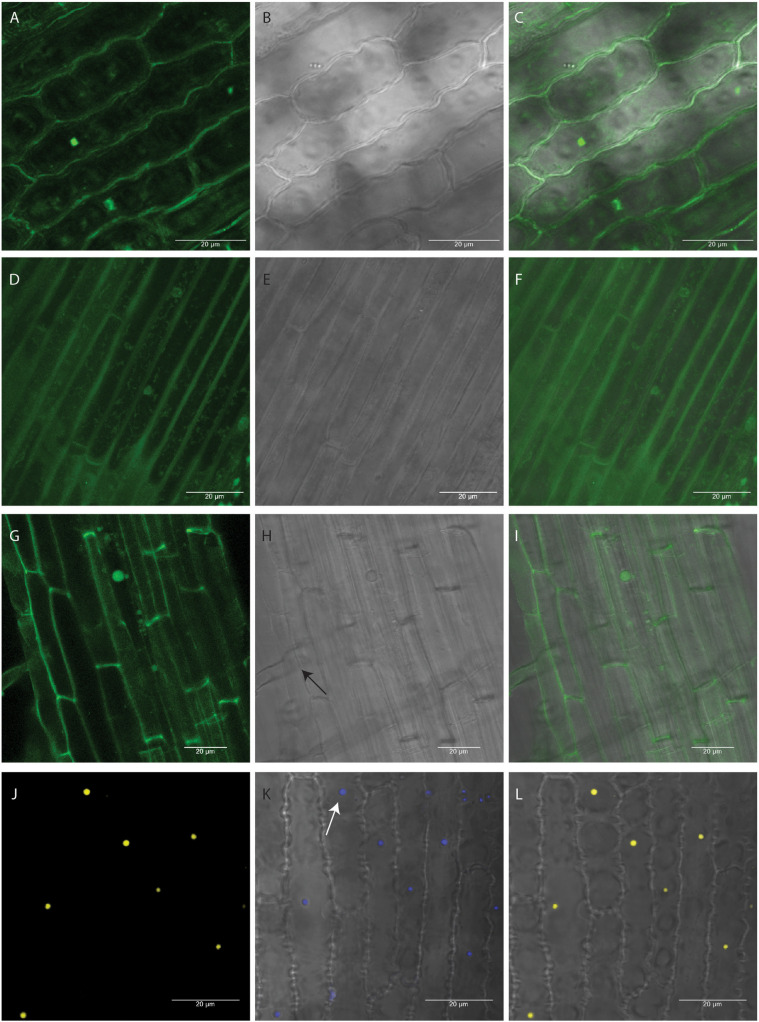
Transient expression of pSITE-2CA (empty GFP vector) construct and BiFc of OsbZIP48 in rice seedling. **(A–C)** GFP localization of pSITE-2CA vector in rice epidermal cells at 63x. **(D–F)** GFP localization of pSITE-2CA vector in rice coleoptile cells at 63x. **(G–I)** GFP localization of pSITE-2CA vector in rice root cells at 63x. Arrow indicates root hair. **(J–L)** BiFc showing homodimerization of OsbZIP48 (using *OsbZIP48* cloned into BiFc vectors pSITE-nEYFP-C1 and pSITE-nEYFP-N1) in nucleus of rice epidermal cells at 63X. **(A,D,G,J)** Show cells under dark field. **(B,E,H,K)** Show same cells under bright field with **(K)** also showing DAPI stained nucleus. Arrow indicates DAPI stained nucleus. **(C,F,I,L)** Show merged picture of the same cells.

It was reported earlier that at 20°C, *Agrobacterium* shows the highest amount of T-pili whereas 28°C strongly inhibits extracellular assembly of the major T-pilus component VirB2 and the pilus-associated protein VirB5 (Baron et al., 2001). Although the seedlings were grown at 20°C after co-cultivation, we wanted to see if there is any effect of the *Agrobacterium* primary culture being grown at 20°C on transformation efficiency. Thus, the *Agrobacterium* culture for co-cultivation was grown at 20°C and the co-cultivation of seedlings in liquid NB medium for 1 h was also performed at 20°C. It was found that as compared to *Agrobacterium* liquid culture grown at 20°C, the culture grown and co-cultivated at 28°C showed higher transformation efficiency (Figure 2C). Among the different *Agrobacterium* densities used, culture with 0.6 A_600_ showed maximum efficiency (Figure 2D). Application of vacuum pressure significantly increased the transformation efficiency as compared to the absence of vacuum (Figure 2E). Among the different concentrations of acetosyringone used, 30 mg/L showed maximum efficiency followed by 90 mg/L (Figure 2F). Plants are known to release phenolic compounds and sugars on wounding, which results in activation of VirG protein that in turn activates *vir* gene promoters (Gelvin, 2003). Thus, different concentrations of glucose were checked for transformation efficiency in co-cultivation medium and it was found that 2% glucose showed maximum efficiency (Figure 2G). Different concentrations of Silwet^®^ L-77 surfactant were also checked as it increases the ability of *Agrobacterium* to bind to the surface of rice seedlings. It was found that 0.02% followed by 0.05% and 0.04% showed good transformation efficiency (Figure 2H). Based on standardization of different components of co-cultivation medium, 6-day-old, light-grown seedlings (at 20°C) were co-cultivated with *Agrobacterium* grown at 28°C and A_600_ 0.6 in NB medium with 3% sucrose containing 0.02% Silwet^®^ L-77, 2% glucose, and 30 mg/L acetosyringone. The co-cultivated seedlings were allowed to grow under light conditions at 20°C for 9 days and the first leaf was visualized under the confocal microscope. Figure 3 gives an overview of the standardized protocol along with troubleshooting tips. To visually verify the transformation efficiency of the protocol, GUS histochemical assay was performed on the transformed rice seedlings. Figure 4 shows the results of GUS assay of first leaf, coleoptile and root of seedlings transformed with pCAMBIA1304 vector. We were able to detect GUS in the root, coleoptile and first leaf of the seedlings with maximum blue color visible in first leaf. In order to validate the effectiveness of the protocol at protein level, western blot was performed from protein extracts of first leaf of the seedlings transformed by pCAMBIA1302 (Figure 4D). A band corresponding to GFP protein size was visible in the protein extracts of first leaf of seedlings transformed by pCAMBIA1302 but was absent in the protein extracts of first leaf of untransformed seedlings, thereby, confirming the expression of genes present in transformed vector in the cells of rice first leaf. The rice seedlings were then transformed by pSITE-2CA vector which contains GFP as fluorescent reporter. The cells expressing GFP were visible in the roots, coleoptile and first leaf of rice seedlings. Figure 5 shows the transient expression of pSITE-2CA vector in the rice leaf epidermal cells (a-c), coleoptile cells (d-f) and root cells (g-i) while homodimerization of OsbZIP48 using BiFc vectors in leaf epidermal cells is shown in Figures 5J–L.

## Discussion

Epicuticular wax and high silica content are the biggest bottlenecks of *Agrobacterium*-based infiltration methods in rice plants as they prevent the *Agrobacterium* from attaching and penetrating the plant surface (Sharma et al., 2020). The source of silica for plants is the water and soil in which the plant grows. Therefore, to reduce the silica content of rice plants, the rice seedlings were grown on Whatman^®^ No.1 paper supplied with Milli-Q water. High epicuticular wax content in rice plants is another challenge. Light and temperature conditions are known to alter the epicuticular wax quantity and composition in *Arabidopsis*, citruses and barley when grown in dark and at low temperature resulting in reduced epicuticular wax content (Giese, 1975; Riederer and Schneider, 1990; Go et al., 2014). IRRI has classified rice growing temperatures into two groups- cool and warm. Temperature between 16 to 25°C is considered to be cool temperature while the temperature between 25 to 35°C is considered as warm (Vergara, 1992). Thus, two temperatures, 20 and 22°C were considered for growing rice seedlings for transient expression analysis as at these temperatures the rice seedlings first leaf emerged within 1 week and the growth temperature was cool (Figure 1). The coleoptile of dark-grown seedlings was larger than the light-grown seedlings and remained alive for a longer duration. For light-grown seedlings, the next fastest emerging tissue was the first leaf. For easier detection of GFP-tagged protein localization, the cells should have less chlorophyll and should be bigger in size like onion epidermal cells. Therefore, the cell structure of dark-grown coleoptile and light-grown first leaf was visualized under the confocal microscope to determine which tissue cells will be better for protein localization experiments. The cells of coleoptile were much larger as compared to the cells of the first leaf. They closely resembled onion epidermal cells and could be easily visualized under low magnification (Figure 1C). The first leaf cells showed epidermal cells with zig-zag cell boundary followed by mesophyll cells. Chlorophyll was present in the light-grown first leaf while the dark-grown coleoptile lacked chlorophyll. The transformation in coleoptile was not very successful due to the short life span of the coleoptile. It takes at least 6–9 days for proper expression of the transformed gene in rice cells at 20°C, by which time the cells of the coleoptile begin to senesce.

The next critical step was to identify the perfect stage of co-cultivation for the first leaf because even with the longer life span, sufficient expression of the transformed gene would be desirable before the leaf begins to senesce. Taking these factors into consideration, the development pattern of the first leaf was studied (Supplementary Table 1). It was found that at day 6, more than 50% of seedlings had their first leaf emerged to a certain length making it the ideal stage for co-cultivation as there will be ample time for expression of transformed genes before the leaf starts to senesce. It is important to note that this development profile is for the Kitaake variety of *japonica* rice and will differ from variety to variety. Every rice variety has a different growth rate and, therefore, if using other varieties, the developmental stage (first leaf has emerged and is at least 0.5 to 1 cm in length) should be considered for co-cultivation and not the day after germination. Some varieties may take longer time to grow at 20°C. In that case 22°C as growth temperature can be tried. We had found that the PB-1 variety of *indica* rice showed very slow growth at 20°C as compared to Kitaake and was not very healthy. It showed better and faster growth when grown at 22°C (data not shown). Therefore, standardization for selection of growth stage for co-cultivation will be required for different rice varieties.

After transformation of rice seedlings, when GUS histochemical assay was done, blue color developed in the roots as well as coleoptile of the seedlings in addition to the first leaf. The intensity of blue color was, however, more in first leaf. Nevertheless, we searched for GFP expressing cells in root and coleoptile under the microscope. We were able to find some GFP expressing cells in the coleoptile but the frequency was less and we also encountered a number of dead cells. The frequency of GFP expressing cells was better in roots but the presence of root hairs and the cylindrical structure of the roots made the visualization little challenging. The first leaf epidermal cells were the easiest to visualize and had high percentage of transformation efficiency. Thus, the study of the localization pattern of a protein in different types of rice cells as well as movement of mobile protein signals from root to shoot or vice versa can be performed using this method.

The current scenario for *in-vivo* intracellular localization and interaction of rice proteins using transient assays mainly involves the use of rice protoplasts or other heterologous systems like tobacco leaves and *Arabidopsis* (Zhang et al., 2011). The use of protoplasts requires a large amount of plasmid DNA and expensive enzymes. Additionally, it does not fully replicate the *in-vivo* conditions of the rice plants. The changes in protein localization and protein-protein interaction in response to various stresses is difficult to study in protoplasts as they are prone to bursting in the absence of ambient conditions (Marx, 2016). Use of heterologous systems like tobacco and *Arabidopsis* have their own drawbacks as they are dicot plants. Rice is a model organism for monocot plants and the absence of an efficient transient transformation system has been the biggest drawback for the progress of research in rice, particularly with respect to elucidating the role of genes with unknown function. The two methods generally used for transient transformation of rice cells are particle bombardment and *Agrobacterium*-mediated transformation. The particle bombardment method requires expensive equipment and chemicals like particle gun and gold particles and is not that efficient (Luginbuehl et al., 2020). The only articles we could find on *Agrobacterium*-mediated transient expression in rice were by Li et al. (2009) and Andrieu et al. (2012). The method described by Andrieu et al. (2012) used mature rice plants grown in the greenhouse for transformation and required an in-house built needle apparatus which not only significantly increases the duration of the protocol but also makes visualization of cells under the confocal microscope more difficult as the rice leaves of mature plants are thicker than the first leaf of seedlings. Further, the article was more focussed on the transient induction of gene silencing in rice leaves as compared to visualization of fluorescent reporters in rice cells. The method described by Li et al. (2009) was mainly for transient expression in *Arabidopsis*. However, they showed that their protocol worked for monocots like rice and sorghum. The transient expression of gus in rice seedlings by their protocol was mainly seen at the base of the seedlings and in the root. Here, we present an efficient transient gene expression protocol that is of short duration (15 days), inexpensive (as it does not require high-end equipment or expensive chemicals), and uses a tissue which is thinner making it easy to visualize protein localization under the confocal microscope. The advantages and disadvantages of transient expression in rice seedlings has been described in Table 1 in detail. Furthermore, the transformed seedlings can be subjected to specialized treatments such as hormone treatment and abiotic stresses much more easily as compared to protoplasts. Thus, we envisage that this method will have a significant positive impact on the status of rice research globally.

**TABLE 1:** Advantages and limitations of transient expression in rice seedlings.

**Advantages**
Transient expression in rice seedling represents the *in-vivo* conditions of intact rice cells.
It does not require expensive enzymes or specialized equipment or large amount of vector DNA like the methods involving protoplast or particle bombardment do.
The problem of protoplast bursting because of changes in osmoticum does not exist in transient expression of rice seedlings.
Localization of proteins in cells of different tissues of rice plant like root, coleoptile, epidermal cells and mesophyll cells of leaf can be studied which is not possible by methods involving protoplasts or particle bombardment.
Changes in protein localization on application of different abiotic stress or hormonal treatment can be studied by this method which is not possible by the method involving protoplast.
The first leaf of rice is thinner as compared to mature leaves and since they are grown at 20°C, the problem of light scattering by thick waxy cuticle which impedes high resolution fluorescent protein visualization is largely absent.
This method can be used to test the efficiency of gRNAs before raising CRISPR/CAS9 stable transgenics.

**Disadvantages**

The first leaf is not flat like a mature leaf and is convex in shape as it encircles the leaf sheath. As a result, problem of focal drift may arise if the tissue is not properly mounted on the slide.
If the seeds are not sterilized properly, infection can be a problem and can hamper healthy growth of rice seedlings.
Since 20°C is not the natural growth temperature, the growth of seedlings is sensitive to the water content in the phyta-jar. Germination rate is also altered with the age of the seeds with fresh seeds germinating faster than old seeds at 20°C.
Slight modifications of co-cultivation stage may be required if using different variety of seeds.

## Conclusion

For years, the presence of thick waxy cuticle and high silica content has been a major barrier in standardizing methods of transient expression in rice plants. As a result, transient assays of rice genes was majorly performed in rice protoplasts or dicots plants like *Arabidopsis*. But none of these represented the exact conditions of intact rice cells. Methods involving particle bombardment of intact rice cells had major limitations since they gave lower transformation efficiency and also required high-end equipment like particle bombardment. Apart from making transformation of cells by *Agrobacterium* more difficult, the thick waxy cuticle also caused light scattering and impeded visualization of fluorescent reporters using confocal microscopy. By growing rice seedlings at low temperature and in presence of only Milli-Q water, we were able to overcome the barriers of waxy cuticle and high silica content. This was because low temperature reduced epicuticular wax production in rice plants and Milli-Q water removed any external source of silica, thereby, reducing its overall content in the rice seedlings. Along with the standardization of co-cultivation medium, a highly efficient method for transient assay in rice seedlings has been achieved and this method could pave the way for accelerating the pace of rice research, bringing it at par with other model plants like *Arabidopsis* and tobacco.

## Data Availability Statement

All datasets generated for this study are included in the article/Supplementary Material.

## Author Contributions

NB designed, performed the experiments, and wrote the manuscript. DC and JPK checked the manuscript. All authors contributed to the article and approved the submitted version.

## Conflict of Interest

The authors declare that the research was conducted in the absence of any commercial or financial relationships that could be construed as a potential conflict of interest.
