# Supplementary material for: A Rapid and Highly Efficient Method for Transient Gene Expression in Rice Plants
Source: Front Plant Sci. 2020 Oct 15;11:584011. doi: 10.3389/fpls.2020.584011 (PMC7593772; doi:10.3389/fpls.2020.584011)
Supplement: Supplementary file 1 [file Table_1.docx]

**Supplementary table 1:** Development pattern of rice seedlings grown at different temperatures and under light and dark conditions.

| 1. **20^°^C dark grown** | | | | | | | | |
| --- | --- | --- | --- | --- | --- | --- | --- | --- |
|  | **4-D** | **5-D** | **6-D** | **7-D** | **8-D** | **9-D** | **10-D** | **11-D** |
| COLEOPTILE | 0.16 cm ± 0.031 | 0.155 cm ± 0.022 | 0.68 cm ± 0.055 | 0.8 cm ± 0.031 | 1.011 cm ± 0.033 | 1.2 cm ± 0.03 | 1.2 cm ± 0.046 | 1.2 cm ± 0.044 |
| 1ST LEAF | 0 | 0 | 0 | 0 | 0 | 0 | 0 | 1.4 cm ± 0.047 |
| Percentage emergence of first leaf | 0 | 0 | 0 | 0 | 0 | 0 | 0 | 39.5% ± 12.4 |
| 1. **20°C light grown** | | | | | | | | |
|  | **4-D** | **5-D** | **6-D** | **7-D** | **8-D** | **9-D** | **10-D** | **11-D** |
| COLEOPTILE | 0.31 cm ± 0.023 | 0.28 cm ± 0.032 | 0.41 cm ± 0.031 | 0.49 cm ± 0.023 | 0.52 cm ± 0.021 | 0.53 cm ± 0.015 | 0.55 cm ± 0.022 | 0.61 cm ± 0.017 |
| 1ST LEAF | 0 | 0.45 cm ± 0.022 | 0.5 cm ± 0.02 | 0.52 cm ± 0.013 | 0.61 cm ± 0.023 | 1.17 cm ± 0.073 | 1.23 cm ± 0.051 | 1.38 cm ± 0.029 |
| Percentage emergence of first leaf |  | 20% ± 12.75 | 60% ± 12 | 98.35 % ± 0.85 | 98.5% ± 0.5 | 99.45% ± 0.25 | 99.55% ± 0.15 | 100% ± 0 |
| 1. **22°C dark grown** | | | | | | | | |
|  | **3-D** | **4-D** | **5-D** | **6-D** | **7-D** | **8-D** | **9-D** | **10-D** |
| COLEOPTILE | 0.11 cm ± 0.01 | 0.36 cm ± 0.026 | 0.85 cm ± 0.037 | 0.97 cm ± 0.036 | 1.56 cm ± 0.09 | 1.76 cm ± 0.126 | 1.93 cm ± 0.316 | 1.95 cm ± 0.142 |
| 1ST LEAF | 0 | 0 | 0 | 0 | 2.25 cm ± 0.156 | 2.3 cm ± 0.132 | 2.5 cm ± 0.140 | 2.82 cm ± 0.095 |
| Percentage emergence of first leaf | 0 | 0 | 0 | 0 | 21.5% ± 5.54 | 62.5% **±** 10.75 | 95% ± 1 | 100% ± 0 |
| 1. **22°C Light grown** | | | | | | | | |
|  | **3-D** | **4-D** | **5-D** | **6-D** | **7-D** | **8-D** | **9-D** | **10-D** |
| COLEOPTILE | 0.18 cm ± 0.02 | 0.36 cm ± 0.026 | 0.51 cm ± 0.017 | 0.52 cm ± 0.026 | 0.53 cm ± 0.037 | 0.54 cm ± 0.024 | 0.55 cm ± 0.04 | 0.58 cm ± 0.032 |
| 1ST LEAF | 0 | 0.43 cm ± 0.018 | 0.58 cm ± 0.04 | 0.84 cm ± 0.049 | 0.9 cm ± 0.045 | 0.96 cm ± 0.047 | 1.47 cm ± 0.042 | 1.53 cm ± 0.03 |
| Percentage emergence of first leaf | 0 | 30% **±** 10 | 84% ± 2 | 96.9% ± 0.7 | 99% ± 1 | 99.5% ± 0.5 | 99.5% ± 0.5 | 100% ± 0 |

n=10 seedlings (mean ± standard error)
