# Supplementary figures and images for: A Rapid and Highly Efficient Method for Transient Gene Expression in Rice Plants
Source: Front Plant Sci. 2020 Oct 15;11:584011. doi: 10.3389/fpls.2020.584011 (PMC7593772; doi:10.3389/fpls.2020.584011)

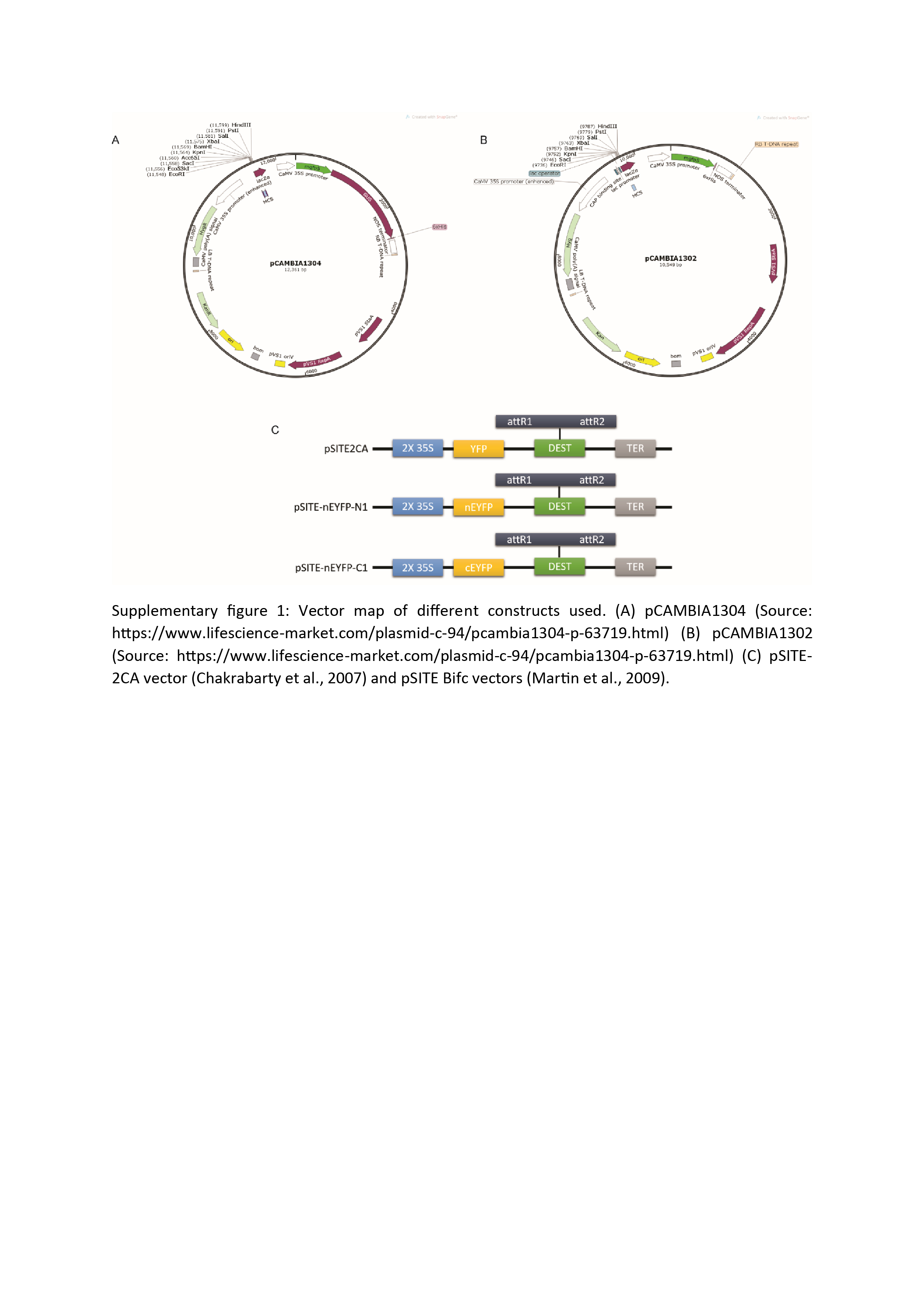

Supplement: Supplementary file 2 [file Image_1.jpg]
